# SASpy: a PyMOL plugin for manipulation and refinement of hybrid models against small angle X-ray scattering data

**DOI:** 10.1093/bioinformatics/btw071

**Published:** 2016-02-28

**Authors:** Alejandro Panjkovich, Dmitri I. Svergun

**Affiliations:** European Molecular Biology Laboratory, Hamburg Outstation, EMBL C/O DESY, Hamburg 22607, Germany

## Abstract

**Summary:** Complex formation and conformational transitions of biological macromolecules in solution can be effectively studied using the information about overall shape and size provided by small angle X-ray scattering (SAXS). Hybrid modeling is often applied to integrate high-resolution models into SAXS data analysis. To facilitate this task, we present SASpy, a PyMOL plugin that provides an easy-to-use graphical interface for SAXS-based hybrid modeling. Through a few mouse clicks in SASpy, low-resolution models can be superimposed to high-resolution structures, theoretical scattering profiles and fits can be calculated and displayed on-the-fly. Mouse-based manual rearrangements of complexes are conveniently applied to rapidly check and interactively refine tentative models. Interfaces to automated rigid-body and flexible refinement of macromolecular models against the experimental SAXS data are provided.

**Availability and implementation:** SASpy is available as open source at: github.com/emblsaxs/saspy/. Working installations of both PyMOL (www.pymol.org) and ATSAS (www.embl-hamburg.de/biosaxs/download.html) are required.

**Contact:** apanjkovich@embl-hamburg.de or svergun@embl-hamburg.de

## 1 Introduction

Small angle X-ray scattering (SAXS) can be used to obtain size, shape and oligomerization information of biological macromolecules in solution, and the method has become an important part of the structural biology toolbox (Graewert and Svergun, 2013). The interpretation of SAXS experiments relies heavily on computational tools, such as the comprehensive collection of programs in the multiplatform ATSAS package (Petoukhov *et al.*, 2012). ATSAS includes programs for SAXS data manipulation and analysis, calculation of overall parameters, *ab initio* shape determination and oligomeric analysis. Given the atomic or predicted models of the entire particle or of its domains/subunits, tools are available to compute theoretical scattering profiles, fits and also to perform hybrid modeling and refinement (Petoukhov and Svergun, 2005). Most of the ATSAS programs operate through the command line interface (CLI), but often a graphical way to interact with models can be convenient, e.g. visually rearranging subunits in a macromolecular complex may be simpler than defining a set of translations and rotations numerically.

At present, there is a point-and-click interface to evaluate theoretical SAXS profiles in the Chimera visualization software (Huang *et al.*, 2014). Chimera’s SAXS module requires an active internet connection (it is based on the FoXS web server (Schneidman-Duhovny *et al.*, 2010)) and it is limited to the computation of theoretical scattering from a given model and its fit to experimental data. Much more elaborated manipulations including rigid-body refinement of complexes are available through the graphical interface of MASSHA (Konarev *et al.*, 2001), which is included in ATSAS. MASSHA incorporates its own three-dimensional (3D) display and refinement functions, but has some limitations. Most notably, it is Windows-only; further, it uses keystrokes instead of the mouse for the manual rearrangement of complexes; finally, MASSHA requires precalculated amplitudes of the individual subunits or domains to perform rigid-body modeling.

To overcome the limitations mentioned above, we present the SASpy plugin that combines tools available in the ATSAS software suite with the graphical display resources of the popular PyMOL molecular visualization software (DeLano, 2015). Given its transparent implementation, SASpy serves as well as a starting point for the user to further automatize or parameterize the analysis workflow through the CLI, if needed. Special care has been taken to make through SASpy a simple and flexible interface to ATSAS that can readily be used without specific training and with minimum necessary input files and parameters. As with other PyMOL plugins, SASpy is distributed as open source. This allows the users to modify or add functions at will, extending the applicability and usefulness of SASpy to fit their particular workflows.

## 2 Implementation

The SASpy main window presents a series of tabs that cover different procedures. Loaded models can be selected right below the tabs, and as with SAXS experimental input files, the selection is preserved and available as the user switches through the different tabs and procedures described below. For most procedures, the corresponding binary from ATSAS is executed with appropriate parameters within a temporary working directory and the results are presented to the user. During the calculations, the output log is displayed in the PyMOL terminal to keep the user up-to-date about the progress, possible errors and available results. Upon completion of the execution of a refinement step the obtained models are loaded into PyMOL and displayed automatically. If SAXS profiles are estimated or fitted, these will be displayed by default using the program SASPLOT (Petoukhov *et al.*, 2012), but the user can set an alternative data display program in the configuration tab.

### 2.1 Basic operation and model manipulation

To calculate the default theoretical scattering of a given model, SASpy will execute CRYSOL (Svergun *et al.*, 1995) in predictive mode and display both the output log and the obtained curve. If the user selects a .dat file containing experimental SAXS data, CRYSOL will be executed in the fitting mode and the obtained fit against the experimental data will be displayed. A generic expansion of PyMOL functionality is provided through the SUPALM tab, which allows one to superimpose high- and low-resolution models. SUPALM is a recently developed faster version of SUPCOMB (Kozin and Svergun, 2001) for automated superposition of two objects without correspondence between individual coordinates (atoms or beads). The matched model is presented graphically and a normalized spatial discrepancy (Kozin and Svergun, 2001) is displayed as a quantitative estimate of similarity between the objects. When working with a macromolecular complex, the subunits can be loaded as independent coordinate files and rearranged (moved or rotated) using the editing features of PyMOL. The fit of the rearranged assembly against the experimental SAXS data can be obtained using the CRYSOL tab. The manual modeling may be useful to get a feeling of the overall sensitivity of the SAXS data to the rearrangements (e.g. for educational purposes) but can also serve as the starting point of the refinement procedures described below.

### 2.2 Hybrid modeling and refinement

Besides model manipulation and analysis, SASpy also offers an interface for automated refinement. Given the SAXS data from a macromolecular assembly of unknown structure, high-resolution models of its subunits, if available, can be used to construct a model of the entire assembly. Automated rigid-body modeling can be carried out in SASpy through the SASREF tab, which allows the user to select a set of models (subunits) and the SAXS profile of the assembly to launch the program SASREF (Petoukhov and Svergun, 2005). Using SASREF through SASpy is much simpler and more intuitive than the standard command line version. Through SASpy, the user does not need to provide precomputed amplitudes of the subunits (which are calculated on-the-fly). Moreover, the positions/orientations of the current configuration shown in the PyMOL screen are used as the starting point for the refinement, obviating the need for the user to specify the numerical values for rotations and translations of the individual subunits as in the CLI version. The same applies to the SREFLEX tab, which utilizes a recently developed approach (Panjkovich and Svergun, 2016) to refine the models consisting of one or multiple high-resolution structures against a SAXS experimental profile by flexible fitting based on normal mode analysis. The refinements by SASREF and SREFLEX are executed as separated CPU threads, so the user can continue to work normally within PyMOL.

## 3 Concluding remarks

SASpy, available for the three major computing platforms (Windows, Mac OS X and Linux), is easy to install as a PyMOL plugin. SASpy requires a working installation of PyMOL and the source code can be downloaded and compiled from sourceforge.net/projects/pymol/. Pre-compiled binaries can be obtained from Schrödinger at www.pymol.org, these are freely available for educational purposes. A local installation of ATSAS version 2.7 or higher (Petoukhov *et al.*, 2012) is also required for correct functionality of SASpy, as it automated superposition and SAS-related features are based on ATSAS binaries. The ATSAS installation package is freely available to academic users at www.embl-hamburg.de/biosaxs/download.html and includes a copy of SASpy. The plugin should be useful for the beginners in SAXS as it serves as a graphical interface to multiple ATSAS functions. We also expect SASpy to be an intuitive educational tool superseding the Windows-only program MASSHA (Konarev *et al.*, 2001). SASpy shall also be useful for experts providing a convenient environment to modify models interactively and allowing rapid comparisons to be executed and displayed on line. SASpy may also be further expanded by the community, given its open source nature.

## References

[btw071-B1] DeLano W. (2015). *The PyMOL Molecular Graphics System*, Version 1.7.4 Schrödinger, LLC.

[btw071-B2] Graewert M.A. Svergun D.I. (2013) Impact and progress in small and wide angle X-ray scattering (SAXS and WAXS). Curr Opin Struct Biol, 23, 748–754. 23835228 10.1016/j.sbi.2013.06.007

[btw071-B3] Huang C.C. (2014) Enhancing UCSF chimera through web services. Nucleic Acids Res., 42, W478–W484. 24861624 10.1093/nar/gku377 PMC4086125

[btw071-B4] Konarev P.V. (2001) MASSHA a graphics system for rigid-body modelling of macromolecular complexes against solution scattering data. J. Appl. Crystallogr., 34, 527–532.

[btw071-B5] Kozin M.B. Svergun D.I. (2001) Automated matching of high- and low-resolution structural models. J. Appl. Crystallogr., 34, 33–41.

[btw071-B6] Panjkovich A. Svergun D.I. (2016) Deciphering conformational transitions of proteins by small angle X-ray scattering and normal mode analysis. Phys. Chem. Chem. Phys., 18, 5707–5719. 26611321 10.1039/c5cp04540a

[btw071-B7] Petoukhov M.V. Svergun D.I. (2005) Global rigid body modeling of macromolecular complexes against small-angle scattering data. Biophys J., 89, 1237–1250. 15923225 10.1529/biophysj.105.064154 PMC1366608

[btw071-B8] Petoukhov M.V. (2012) New developments in the *ATSAS* program package for small-angle scattering data analysis. J. Appl. Crystallogr., 45, 342–350. 25484842 10.1107/S0021889812007662 PMC4233345

[btw071-B9] Schneidman-Duhovny D. (2010) FoXS: a web server for rapid computation and fitting of SAXS profiles. Nucleic Acids Res., 38, W540–W544. 20507903 10.1093/nar/gkq461 PMC2896111

[btw071-B10] Svergun D. (1995) CRYSOL a program to evaluate X-ray solution scattering of biological macromolecules from atomic coordinates. J. Appl. Crystallogr., 28, 768–773.

